# Sarcopenia and Its Relationships with Depression, Cognition, and Physical Activity in Thai Community-Dwelling Older Adults

**DOI:** 10.1155/2020/8041489

**Published:** 2020-12-22

**Authors:** Kornanong Yuenyongchaiwat, Rumpa Boonsinsukh

**Affiliations:** ^1^Physiotherapy Department, Faculty of Allied Health Sciences, Thammasat University, Rangsit Campus, Bangkok, Thailand; ^2^Faculty of Physical Therapy, Srinakharinwirot University, Ongkharak Campus, Nakhon Nayok, Thailand

## Abstract

**Background:**

Age-related sarcopenia is associated with physical decline, including poor functional capacity, lack of physical activity, problems with activities of daily living, and disability. However, little is known about the association between mental health problems and cognitive function in older adults with sarcopenia. Therefore, this study explored community-dwelling older adults' sarcopenia prevalence and related associations with depression, cognitive performance, and physical activity.

**Methods:**

This cross-sectional study included 330 community-dwelling older adults (66.85 ± 5.54 years, 76.06% female). Based on the Asian Working Group for Sarcopenia guidelines, gait speed, muscle mass, and handgrip were assessed. All participants responded to a set of questionnaires (e.g., Global Physical Activity Questionnaire, cognitive assessment, and depression scale). Logistic regression analysis and multivariate logistic regression were used to determine independent predictors for sarcopenia.

**Results:**

Overall, 16.1% of the participants were identified as having sarcopenia. Further, advanced age (i.e., mean age ≥ 70 years; odds ratio: 4.67), high depression scores (odds ratio: 2.09), mild cognitive impairment (odds ratio: 0.22), and low physical activity levels (odds ratio: 1.96) were significant associated risk factors for sarcopenia after adjusting for age, sex, and educational level.

**Conclusions:**

Sarcopenia can lead to adverse health outcomes (i.e., depressive symptoms, cognitive decline, and low physical activity) in older adults.

## 1. Introduction

Globally, the prevalence of age-related health problems is becoming a major concern as the proportion of older people has markedly increased. It is known that older adults are exposed to a significantly higher risk of developing psychological and cognitive dysfunctions. Additionally, there is growing evidence that depression, decreased physical activity, and cognitive decline are common in older adults and that these disorders are jointly associated [1, 2]. Depressive symptoms are also negatively associated with physical activity. For example, several studies have demonstrated that older adults who have low levels of physical activity show high levels of depression [3, 4]. Further, during long-term follow-up, the association between low levels of physical activity and functional limitations was indicated as a risk factor for physical disabilities, including sarcopenia [5, 6]. Therefore, the reduction of cognitive and physical performance is a common factor resulting in frailty and disability] [7].

Sarcopenia, which occurs in older adults, is defined as a reduction in lean body mass and muscle strength and low physical performance. It is associated with physical decline, including poor functional capacity, risk of falls, problems with activities of daily living, and disability, and also negatively affects mental health outcomes [8, 9]. Evidence has supported an association between sarcopenia and depression; participants with sarcopenia showed increased depressive symptoms [10, 11]. Thus, sarcopenia is becoming a serious health problem worldwide [12].

Older adults often have multiple risk factors for both physical and mental health conditions which can lead to poor quality of life. Although sarcopenia and risk factors for physical and mental health conditions have been reported in several studies, associations between sarcopenia and depression, cognition, and physical activity have not been taken into serious consideration. Therefore, this study aimed to explore the relation between sarcopenia and depression, cognitive performance, and physical activity in community-dwelling older adults.

## 2. Materials and Methods

The cross-sectional study used convenience sampling within a population of male and female community-dwelling older adults aged ≥60 years. Previously, Khongsri et al [13] found the prevalence of sarcopenia to be 30.5% in community-dwelling older adults in Thailand. Therefore, we determined the sample size necessary in the present study to be 326, for 5% precision with a 95% confidence level. The sample size formula in this study was *N* = *Z*^2^*P* (1−*P*)/*d*^2^, where *Z* is the *Z*-statistic for a confidence level of *Z* = 1.960, *P* is the expected prevalence, and *d* is the precision level (*d* = 0.05). To account for incomplete or missing data, the sample size was increased to 330 older adults. Participants were recruited from people living in the local community through poster advertisements and personal contact (i.e., word of mouth). All participants were informed about the study's purpose and procedures, after which they provided written informed consent. The study was approved from the Ethics Human Committee of Thammasat University, based on the Declaration of Helsinki, the Belmont Report, CIOMS Guidelines, and the International Practice (ICH-GCP) COA no. 023/2562. The clinical trial registration is TCTR20190218002.

Participants met the inclusion criteria if they were aged ≥60 years and able to understand and communicate in Thai language. Both males and females were included. Those who had an amputation, were unable to walk, or had visual or hearing impairment that would affect the test results were excluded from the study. Individuals with a history of diagnosed psychiatric problems, medical treatment for the same, and the presence of severe cognitive impairment and/or dementia were also excluded.

### 2.1. Assessment of Sarcopenia

The Asian Working Group for Sarcopenia (AWGS) defines sarcopenia as having low muscle mass plus poor physical performance and/or decreased muscle strength [14].

Body weight and skeletal muscle mass were measured using a bioelectrical impendent analysis (BIA) device (Omron HBF-375 body composition monitor; Omron Healthcare Co., Ltd., Japan). Participants were asked to provide a urine sample prior to the test. During the test, participants were required to stand on two metallic electrodes and hold metallic grip electrodes. The lean body mass was calculated by the percentage of the total body skeletal mass multiplied by body weight and divided by 100. The skeletal muscle mass index (SMI) was computed as follows: skeletal muscle mass divided by height squared (kg/m^2^). Low muscle mass was defined as having an SMI below 7.0 kg/m^2^ for men and below 5.7 kg/m^2^ for women [14].

A handgrip model TKK 5101 (Japan) was used to measure muscle strength. Participants performed the measure three times and the highest of the three measurements was recorded. According to AWGS, the cutoff values in men and women are 26 kg and 18 kg, respectively [14].

Regarding gait speed, participants were asked to walk a distance of 6 meters. Participants performed this action three times, and the average values were calculated for gait speed. The cutoff used for low physical performance was ≤0.8 m/s [14].

### 2.2. Assessment of Depressive Symptoms, Physical Activity, and Cognitive Performance

The Thai Geriatric Depression Scale (TGDS) is a standard depression screening tool. The total TGDS scores range between 0 and 30 points, and the presence of depressive symptoms is determined by a cutoff score of 12; higher scores are interpreted as higher levels of depression. The accuracy of a screening tool for depression was 0.94 and 0.91 for women and men, respectively [15,16].

The Global Physical Activity Questionnaire (GPAQ) was developed by the World Health Organization, and it has been used in several countries to determine physical activity levels [15]. High levels of total physical activity are defined as ≥1,500 MET minutes per week, and low physical activity is < 600 MET minutes per week [17].

The Thai version of the Montreal Cognitive Assessment (MoCA-T) [18, 19], as translated by Hemrungrojn, was used to screen cognitive performance. The scale has been validated, and the internal consistency and sensitivity and specificity of the MoCA-T were found to be 0.914 and 0.8, respectively [20]. A total score of 25 or less is interpreted as mild cognitive impairment (MCI) [20].

Body mass index (BMI) was calculated as body weight (kg) divided by body height squared (m^2^). Data were verified for normality of distribution using the Kolmogorov–Smirnov goodness-of-fit test. Additionally, *t*-tests and chi-square tests were used to compare variables between participants with sarcopenia, where appropriate. Further, logistic regression analysis and multivariate logistic regression were used to determine independent predictors of sarcopenia. A *p* value of <0.05 was considered statistically significant. All statistical analyses were performed with SPSS version 23.

## 3. Results

**Figure 1:**
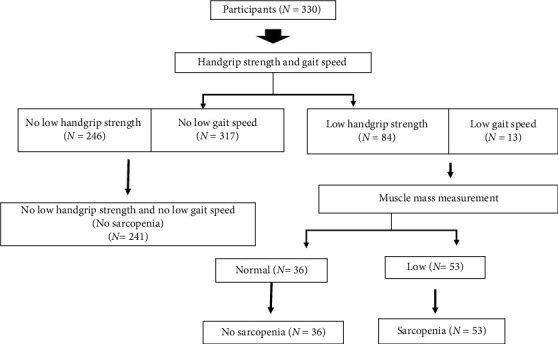
Sarcopenia category by diagnostic algorithms of Asian Working Group for Sarcopenia.

Of the 330 older adults (mean age = 66.85 ± 5.54 years) who participated in the sarcopenia screening, 251 (76.06%) were female. According to the AWGS algorithm, 53 (16.07%) community-dwelling older adults in the present study were identified as having sarcopenia (Figure 1). Specifically, 49 (14.85%) participants were identified as sarcopenic due to poor grip strength (*n* = 48, 14.55%) or slow gait speed (*n* = 1, 0.30%), whereas 4 (1.21%) had a concomitant presence of reduced muscle strength and slow gait speed.

Further, regarding sex, it was found that male participants seem to have a higher rate of sarcopenia than female participants (18.99% vs. 15.14%). However, this difference was not significant (*p*=0.482). Data on the characteristics of the entire study population with sarcopenia are displayed in Table 1. There were significant differences in TGDS scores, MCI, and physical activity levels between older adults with and without sarcopenia. In participants with sarcopenia, cognitive performance and physical activity were lower than in the nonsarcopenic group (*p*_*s*_ < 0.01), and TGDS scores were higher than in the nonsarcopenic group (*p* < 0.001).

Age, sex, educational levels, physical activity levels, cognition, and depressive symptoms were analyzed using logistic regression analysis (see Table 2). Advanced age (i.e., mean ≥ 70 years; odds ratio (OR): 4.67; 95% confidence interval (CI): 2.42–8.24), high TGDS scores (OR: 2.34; 95% CI: 1.22–4.45), MCI (OR: 0.23, 95% CI: 0.05–0.96), and low physical activity levels (OR: 2.51; 95% CI: 1.34–4.56) were significant risk factors for sarcopenia in older adults in the unadjusted analysis. It has been previously reported that differences in age, sex, and educational levels might partially explain low physical activity, depression, and cognitive impairment [2]. Therefore, we conducted a multivariate analysis adjusted for potential confounding variables (i.e., age, sex, and educational levels) and found independent associations with sarcopenia risk for physical activity, depression, and MCI (ORs = 1.96, 2.09, and 0.22, respectively; *p*_*s*_ < 0.05).

## 4. Discussion

The present study focused on the prevalence of sarcopenia according to AWGS criteria, which is based on gait speed, handgrip strength, and muscle mass. Additionally, the associations of sarcopenia with depression, cognitive function, and physical activity were explored. The results indicated that 16.1% of the community-dwelling older adults in our sample population had sarcopenia. Compared to older adults without sarcopenia, those with sarcopenia tended to be of an advanced age and had high levels of depressive symptoms, MCI, and low physical activity levels.

Based on the definitions provided by the AWGS, 16.1% of community-dwelling individuals aged ≥60 years in the present study meet the criteria for sarcopenia. This is comparable to previous studies conducted in Japan, China, and Taiwan, in which the prevalence ranged between 13.4 and 17.9% in both males and female groups [21–23]. However, in contrast to a previous study, the prevalence of sarcopenia was 30.5% in our study's population [13]. This might be because three-fourths of our participants were aged <70 years and only 10 participants were aged ≥80 years, whereas Khongsri et al. [13] recruited participants aged > 60 years and only half were <70 years. Furthermore, the reported prevalence of sarcopenia has been observed to have a wide range, from 8 to 40%, and these findings tend to be based on different diagnostic criteria and living communities (i.e., individual attributes), which might lead to different prevalence rates for sarcopenia [24, 25].

A higher prevalence of sarcopenia was observed in male compared to female participants, which corresponds to other studies conducted in both Asian and European countries [13, 26]. A previous study conducted in Thailand found the prevalence rate to be 33.9% in male participants and 29.3% in female participants [13]. Further, advanced age is also related to sarcopenia risk—the older the age group, the higher the prevalence of sarcopenia. Castillo, Goodman-Gruen, and Kritz-Silverstein [27] reported that the prevalence of sarcopenia increased from 7% in people aged 70–75 to 29% in people aged 85 and older. It was found that the prevalence of sarcopenia was as high as 33.6% in people aged 70 years or older [28]. Therefore, the present study was in line with other studies regarding factors of age and sex, especially the higher prevalence in male older adults. However, it should be noted that female participants were relatively younger; therefore, future studies need to recruit both male and female participants from different age groups. Additionally, different diagnostic criteria and measurements of muscle mass might account for differences in reported prevalence rates for sarcopenia.

Regarding cognition, the present study found that older adults with sarcopenia had higher levels of cognitive impairment compared to those without sarcopenia. Similarly, sarcopenia has been found to be significantly related to cognitive decline in community-dwelling Taiwanese [22] and Japanese older adults [29] and older Korean women [30]. In a systematic review and meta-analysis, it was reported that in seven cross-sectional studies comprising 5,994 individuals in total, a positive relationship between sarcopenia and mild cognitive impairment was originally shown for OR of 2.926 (95% CI: 2.297–3.728) and 2.246 (95% CI: 1.210–4.168) after adjusting for age, sex, educational level, depression, activities of daily living, and physical performance [31].

The results of this study showed depression to be independently associated with sarcopenia in community-dwelling older adults, and this relationship remained after adjusting for age and sex. A relationship between depression and sarcopenia has also been reported in several studies [30, 32]. Lee et al. [30] found an inverse relationship between cognitive performance and depression scores in older Korean women. Further, a systematic review and meta-analysis comprising 15 observational studies indicated that patients with sarcopenia were likely to have depression [10]. Further, Yuenyongchaiwat et al. [2] reported that older adults with cognitive impairment had lower physical activity levels and higher depression levels, compared to individuals without cognitive impairment. Therefore, depression is associated with poor cognitive performance, which often relates to insufficient physical activity or lack of exercise, which could be a contributing factor to sarcopenia. It appears that much of the association with the prevalence of sarcopenia could be prevented or reduced by lifestyle modifications, such as increased exercise or other physical activity. A systematic review of 10 studies found sarcopenia to be associated with low physical activity levels; therefore, increased physical activity or exercise-based interventions could potentially reduce the risk of immobility and also decrease risks related to sarcopenia (e.g., handgrip strength, muscle mass, and physical performance) [6].

Depression could be a contributing factor to sarcopenia; however, sarcopenia could also contribute to increased depression. Additionally, physical inactivity might be linked to both depression and sarcopenia. A meta-analysis of 24 studies reported that interleukin-6 (IL-6) and tumor necrosis factor-*α* (TNF-*α*) were found in higher concentrations in individuals with depression, compared with control participants [33]. It has been reported that inflammatory markers such as TNF-*α* and IL-6 play a key role in the occurrence of sarcopenia and these inflammatory markers are also related to depressive symptoms [34]. Kim et al. [35] found plasma levels of TNF-*α* and IL-6 were negatively associated with cognitive impairment (as defined by the Mini-Mental State Examination). Therefore, activation of the inflammatory response system or inflammatory mediators might be a possible explanation for the associations among sarcopenia, depression, cognitive impairment, and physical activity.

As discussed above, mechanisms linking pathophysiological interrelated factors (e.g., depression, cognition, and physical activity) to sarcopenia are unclear. Sarcopenia has been associated with changes in protein synthesis and degradation, proteolysis, neuromuscular function, and muscle fat content [36]. These associations can lead to negative physical health outcomes, such as physical inactivity, immobility, and poor nutrition.

Accordingly, future studies should examine the mechanisms underlying the development of sarcopenia in larger samples, with follow-up to further clarify the development of sarcopenia and other potential mechanisms. Musumeci [37] reported that decreased muscle strength can also be caused by different problems such as diabetes mellitus, decreased physical activity, high weight, poor nutrition, and loss of muscle mass and strength. Additionally, a new concept regarding the mechanisms underlying sarcopenia is “metaflammation,” which has been described as a metabolic inflammation with metabolic diseases, such as diabetes mellitus and muscle mass [38, 39]. Therefore, one mechanism linking depression, cognitive impairment, and physical activity which are interrelated factors to loss of muscle mass and sarcopenia is inflammatory mediators.

The present study simultaneously assessed depressive symptoms, cognitive performance, and physical activity levels in community-dwelling older adults. However, this study has some limitations. First, the data were collected from only one province in Thailand and therefore might not represent other populations of older adults. Second, this study did not examine factors such as inflammatory markers' levels (e.g., IL-6 and TNF-*α*), C-reactive protein (CRP), and *α*1-antichymotrypsin (ACT). Therefore, future studies are needed to explore the mechanism underlying the development of sarcopenia in older adults.

## Figures and Tables

**Table 1:** Comparisons of demographic data among the nonsarcopenic and sarcopenic elderly.

	Nonsarcopenia (*N* = 277)	Sarcopenia (*N* = 53)	Total (*N* = 330)	95% CI	*p* value
Age (years)	66.14 ± 5.03	70.58 ± 6.52	66.85 ± 5.54	−6.01 to −2.88	<0.001

Sex^*∗*^					0.127
Female	222 (67.3%)	29(8.8%)	251 (76.1%)		
Male	64 (19.4%)	15 (4.5%)	79 (23.9%)		

Education^*∗*^					0.012
Primary school	208 (63.0%)	48 (14.5%)	256 (77.6%)		
Higher than primary school	69 (20.9%)	5 (1.5%)	74 (22.4%)		
BMI (kg/m^2^)	26.19 ± 4.26	22.22 ± 3.51	25.55 ± 4.39	2.74 to 5.19	<0.001
Physical activity (MET^*∗*^min^*∗*^wk^−1^)	3033.54 ± 3890.71	945.66 ± 1869.69	2698.21 ± 3720.35	1012.58 to 3163.17	<0.001
Depression (scores)	7.51 ± 5.29	12.04 ± 6.20	8.23 ± 5.69	−6.14 to −2.93	<0.001
Cognitive function	18.37 ± 4.58	16.60 ± 3.99	18.09 ± 4.53	0.44 to 3.09	0.009

^*∗*^Analysis by chi-square test.

**Table 2:** Logistic regression analysis for risk factors of sarcopenic elderly.

Risk factors for sarcopenia	Odds ratio (95% CI)	*p* value	Adjusted odds ratio (95% CI)	*p* value
Sex	Reference group: female			
Male	1.314 (0.679 to 2.541)	<0.001		

Age	Reference group: 60–69			
≥70	4.465 (2.420 to 8.238)	<0.001		

Education	Reference group: higher education			
Primary school/lower	0.314 (0.120 to 0.820)	0.012		

Physical activity (GPAQ)	Reference group: moderate to high physical activity			
<600 (MET^*∗*^min^*∗*^wk^−1^)	2.511 (1.383 to 4.558)	<0.001	1.962 (1.045 to 3.683) ^$$^	0.036

Depression	Reference group: normal			
Symptoms of depression	2.335 (1.224 to 4.453)	<0.001	2.089 (1.057 to 4.130) ^$$^	0.034

Cognitive performance	Reference group: normal cognition			
Mild cognitive impairment	0.226 (0.053 to 0.963)	0.044	0.215 (0.047 to 0.987) ^$$^	0.048

^$$^Adjusted for age, sex, and educational levels.

## Data Availability

The datasets analyzed during the current study are available from the corresponding author upon reasonable request.
